# Electron beam induced removal of PMMA layer used for graphene transfer

**DOI:** 10.1038/s41598-017-18444-1

**Published:** 2017-12-22

**Authors:** B. H. Son, H. S. Kim, H. Jeong, Ji-Yong Park, Soonil Lee, Y. H. Ahn

**Affiliations:** Department of Physics and Department of Energy Systems Research, Ajou University, Suwon, 16499 Korea

## Abstract

We demonstrate the development of an effective technique to remove the poly methyl methacrylate (PMMA) layer used for transferring graphene synthesized by a chemical vapor deposition (CVD). This was achieved utilizing electron-beam bombardment and following developing processes, prior to the use of conventional organic solvents. Field-effect transistors were fabricated on the transferred graphene in order to explore their Dirac points and carrier motilities in the ambient condition - the results were then compared with those from the conventional wet chemical treatment. It was found that the Dirac points were located close to the zero gate bias when compared to those from the acetone and the acetic acid treatments. Most significantly, the field-effect mobility reached as high as 6770 cm^2^/Vs and 7350 cm^2^/Vs on average for holes and electrons, respectively, which is more than seven times improvement in comparison to conventional acetone treatments for CVD-grown graphene devices.

## Introduction

Two-dimensional materials, such as graphene, have attracted significant interest for their unique electrical, mechanical, and optical properties, and therefore, have a potential use in various fields such as field-effect transistors (FETs)^1–4^, sensors^5,6^, integrated electronic circuits^7,8^, large-scale transparent electrodes^9,10^, and optoelectronics^11,12^. Graphene was first obtained by mechanical exfoliation from graphite, and, latterly, by chemical vapor deposition (CVD) using a variety of metals such as Copper (Cu), Nickel (Ni)^13^ - CVD is the most reliable synthesis method and has the advantage of high quality and high productivity on an industrial scale. In particular, the graphene grown by CVD on a Cu foil is widely used because it is cheap and the growth of a single layer graphene is relatively easy^13^.

In order to fabricate the FETs, graphene grown on Cu foil, using the CVD method, is required to be transferred to the Silicon (Si) substrate^14–18^. This is typically performed using a supporting layer such as poly methyl methacrylate (PMMA) and after the transfer process, the PMMA on the graphene surface will be cleaned using an organic solvent such as acetone^19^. However, because of the strong interaction between the PMMA and the graphene, its residues will inevitably be left at the graphene surface, even after the cleaning processes^20,21^. Alternative transfer techniques have been proposed to achieve high-quality graphene as summarized in the recent literature^22^; however, most of them suffer from residues or degradation during the removal procedures of the supporting layers. Graphene is extremely sensitive to adsorbates and molecules in contact with its surface, hence, the residues tend to act as a dominant source of doping and scattering of charge carriers, degrading the electrical properties of graphene^23^. Therefore, the thorough removal of the PMMA residues is crucial for improving the electrical and optoelectronic characteristics of graphene devices. Various wet chemical treatments such as acetic acid, chloroform, and formamide solution have been proposed to reduce these residues, as an alternative to the conventional acetone solution^24–26^. More recently, advanced techniques have been introduced, including thermal annealing^8,27^, current induced annealing^28^, laser cleaning treatment^29^, Oxygen (O_2_) plasma treatment^30,31^, and ultraviolet (UV) ozone treatment^32–34^, However, they contain rather complicated processes and some of them suffer from the lack of reproducibility. Moreover, their field-effect mobility remains mostly at 3000–4000 cm^2^/Vs which has been the upper limit of the devices fabricated from the CVD-grown graphene so far^35^.

This research proposes a new approach for the effective removal of the PMMA that has been used for transferring the graphene onto silicon substrates, based on the fact that electron bombardment is best suited for removing the PMMA by its nature. The FET devices based on the transferred graphene were fabricated, with which the Dirac points and the field-effect motilities were measured and then compared with the devices from the conventional removal techniques. It was found that the e-beam treatment (ET) provided the superior device performance relative to those found in the literatures.

## Results and Discussion

**Figure 1:**
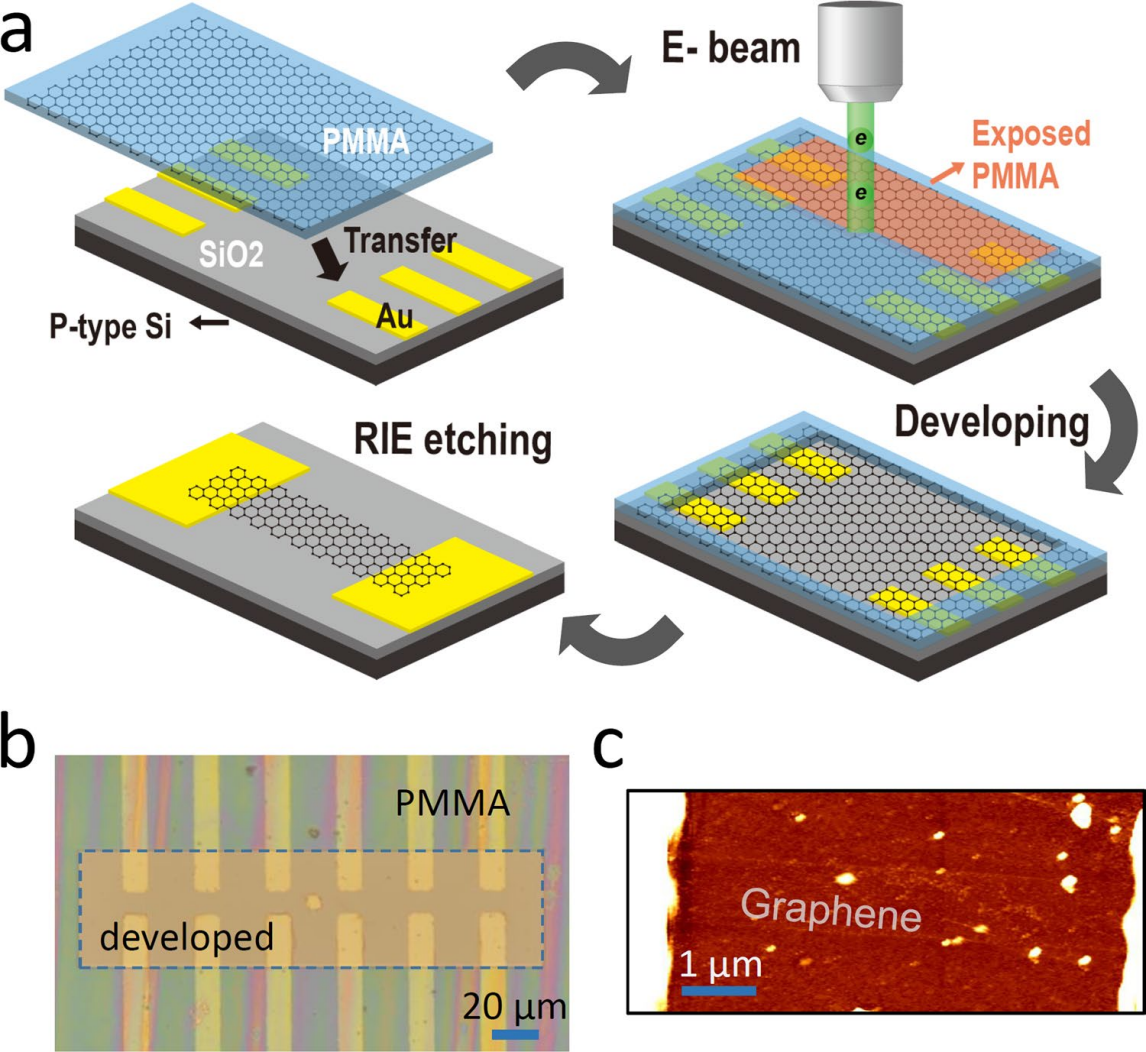
(**a**) Schematic of fabricating graphene FET devices based on the e-beam bombardment technique for removing PMMA layer used for transferring graphene. (**b**) Optical image of graphene FET devices with the part of PMMA layer removed by the e-beam processes (scale bar: 20 μm). (**c**) AFM image of a graphene FET device.

A schematic of the experimental approach used in this research is illustrated in Fig. 1(a). Graphene was prepared utilizing the CVD method on a Cu foil using methane and hydrogen gases. PMMA was employed as a graphene carrier to transfer graphene film from the Cu foil onto a Si substrate with the predefined drain and source electrodes (Cr/Au), fabricated with conventional lithography techniques^36–38^. After the Cu foil was fully dissolved in an ammonium persulfate solution, the PMMA/graphene film was transferred onto the silicon substrate.

As previously mentioned, one of the key elements for fabricating high-performance FET devices is removing the PMMA layer that was used for carrying the graphene. In this research, the PMMA layer was removed using electron-beam bombardment (prior to the chemical treatment), followed by the developing processes. The condition for the e-beam irradiation was same as conventional lithographic procedures, except that the exposure area was relatively large, with a large beam current, enabling us to remove the PMMA residues over many devices, in a relatively short time. An electron-beam, with the areal dose of 100 μC/cm^2^ and current of 500 pA at 25 kV, was irradiated for 20 s through the region (50 × 200 μm^2^) that encompasses more than 6 devices. The exposed region of the PMMA was then removed by the developing process, which featured methyl isobutyl ketone (MIBK) 3:1 solution and lasted for 3 minutes (resultant microscopic image is shown in Fig. 1(b)). After the developing, PMMA residues were removed thoroughly by dipping the sample in acetone solution for 2 hours. We note that the e-beam procedure has to be carried out prior to chemical treatment, because, once the localized PMMA residues are formed during the chemical treatment, it will be more difficult to remove the residues without causing damage to the graphene. Finally, graphene patterns were generated (width ~1 µm and channel length of 7–10 µm) using the electron-beam lithography technique, followed by reactive ion etching as shown in the atomic-force microscopy (AFM) image of Fig. 1(c).

To elucidate the effect of the ET proposed in this research, two different processes were added for comparison – acetone overnight (AO) and acetic acid (AA) methods^19,23,24^. These methods have proven to be very effective in removing the PMMA residue. For the AO process, the sample was submerged in the acetone solution for 24 hours at room temperature. Conversely, during the AA process, the sample was dipped in acetone solution for 1 hour and then immediately immersed in acetic acid for 24 hours at room temperature.

**Figure 2:**
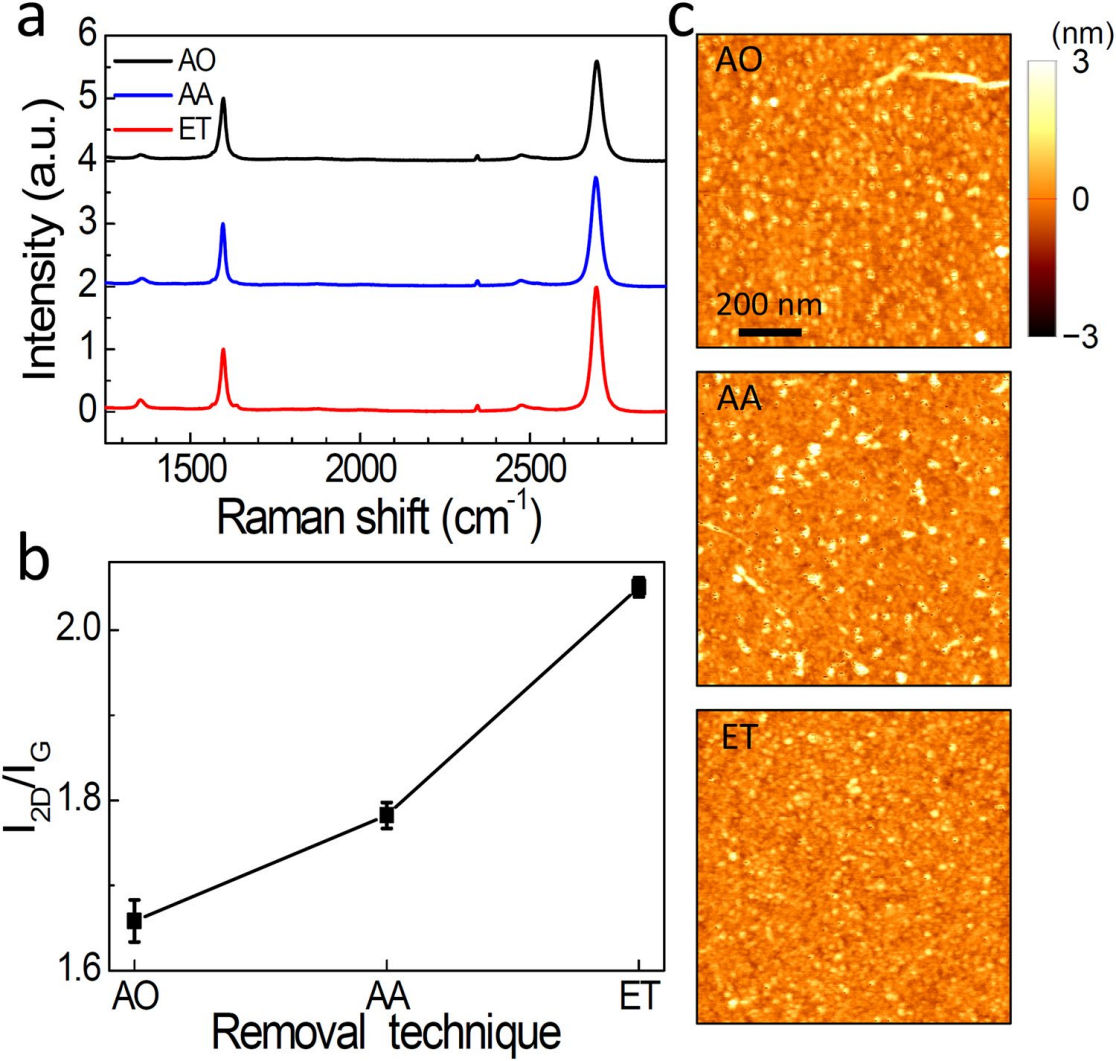
(**a**) Raman spectra for the graphene film in which the PMMA layer was removed by acetone overnight (AO), acetic acid (AA), and e-beam treatment (ET), from top to bottom. (**b**) Intensity ratio of *I*_2D_/*I*_G_ for different PMMA removal techniques. (**c**) AFM images for the graphene surface with different PMMA removal techniques (scale bar: 200 nm).

In Fig. 2(a), we demonstrate Raman spectra of the three different graphene films transferred by AO, AA, and ET processes. We used the laser source at 532 nm for the Raman measurements^19^. The spectra exhibit two distinct peaks at 1597 cm^−1^ (G) and 2695 cm^−1^ (2D), whereas the D peak at 1352 cm^−1^ is suppressed. Clearly, the intensity of 2D peak varied with different PMMA removal techniques, whereas we could not observe a noticeable frequency shift relative to each other. As summarized in Fig. 2(b), the intensity ratio of *I*_2D_/*I*_G_ is highest for ET process, yielding 2.05 on average, as compared to the AO (1.66) and AA (1.78) processes. In general, 2D intensity is suppressed in the presence of PMMA residues at the graphene surface (Supplementary Information S1). This is because the PMMA residues induces the p-type doping effect in graphene^39^ and *I*_2D_/*I*_G_ depends strongly on the carrier density (or the Fermi energy) in graphene^19,40^. Therefore, the increase in *I*_2D_/*I*_G_ is a strong indication that the PMMA residues have been removed more effectively though our novel technique. We also note that *I*_D_ did not change significantly with the ET procedure, indicating that it did not induce the noticeable defect in graphene. This was also consistent with the root-mean-square surface roughness (*R*_q_) characterized from the AFM images^21,41^. As shown in Fig. 2(c), the number of particles (white dots) decreased significantly with the ET procedure as compared to those of AO and AA procedures. The *R*_q_ for ET sample is 0.336 nm, which is lower than AO (0.448 nm) and AA (0.476 nm) cases. We also tested with more than 10 samples, yielding the average *R*_q_ values of 0.380, 0.421, and 0.446 nm, respectively for ET, AA, and AO samples.

**Figure 3:**
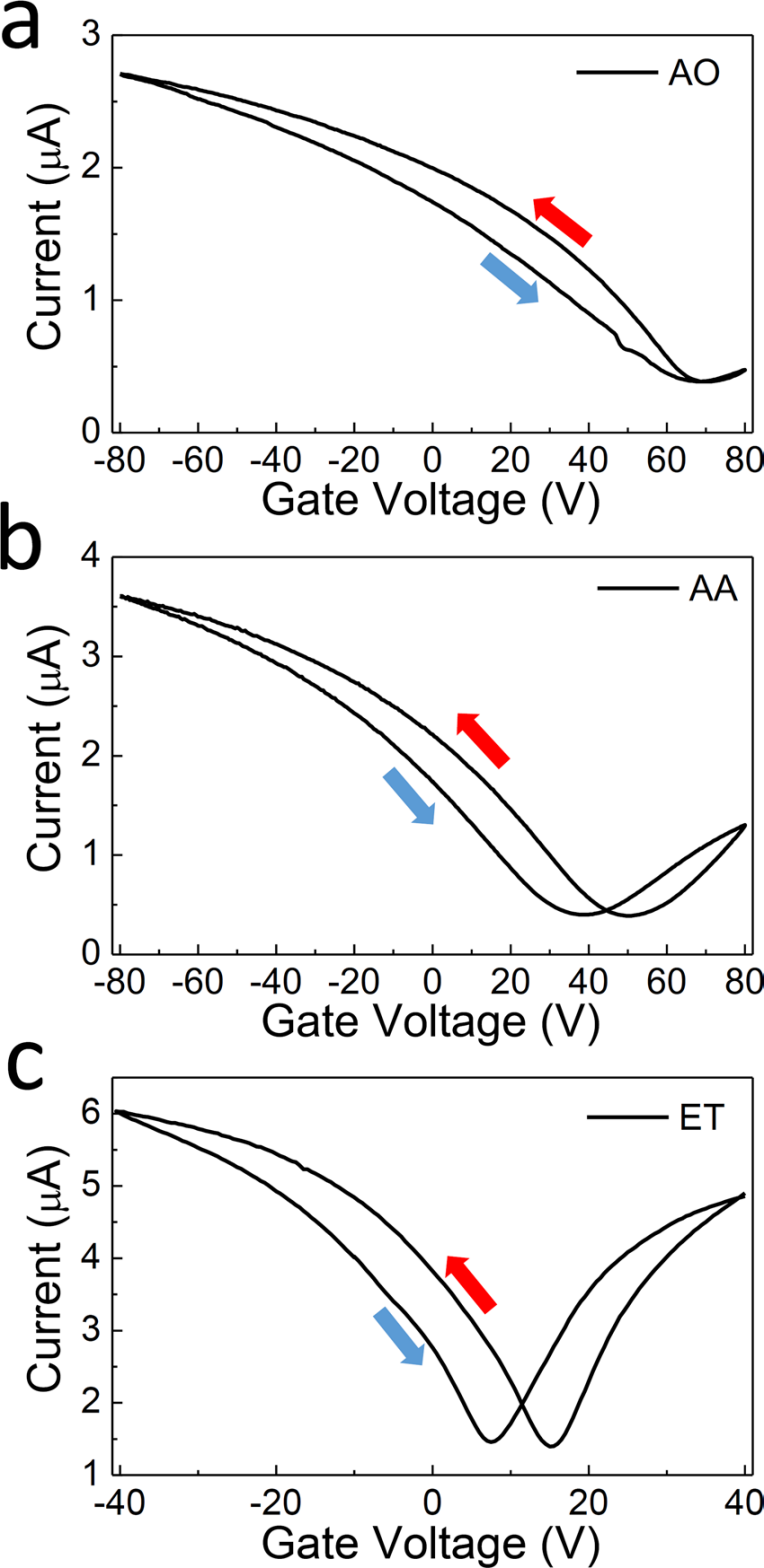
*I*-*V*_G_ characteristics as a function of *V*_G_ at *V*_DS_ = 10 mV for the graphene FET devices in which the PMMA layer was removed by (**a**) acetone overnight (AO) (**b**) acetic acid (AA), and (**c**) e-beam treatment (ET).

The *I*-*V*_G_ characteristics of the devices were measured in the back-gate geometry for the three different PMMA removal procedures as shown in Fig. 3, with the drain-source bias was fixed at *V*_DS_ = 10 mV. For devices with AO and AA processes, the gate voltage (*V*_G_) was swept from −80 V to 80 V, with sweep speed of 5 V/s, and for ET processes, the gate voltage was swept from −40 V to 40 V, again with a sweep speed of 5 V/s. All measurements were taken in the ambient condition. For all of the devices, the switching behavior with the clear Dirac point located in the region of −80 V < *V*_G_ < 80 V could be identified. The Dirac point of the AA sample was found at *V*_G_ = 44.5 V, which is closer to *V*_G_ = 0 V in comparison to that of the AO treated device, which was located at *V*_G_ = 68.0 V. At this point, an average value was evaluated for the two Dirac points obtained with different sweeping directions. The shift in Dirac points for AA devices, in comparison to AO devices, is likely to be due to the reduction in PMMA-induced doping effects, considering that PMMA residue causes p-type doping in the graphene as mentioned above^39^. Importantly, the Dirac point approaches closer to *V*_G_ = 0 V for ET samples, as shown in Fig. 3(c), and yields *V*_G_ = 11.3 V, on average. This is a strong indication that a greater amount of PMMA was removed using e-beam bombardment and the following developing procedures, ultimately proving the usefulness of the approach proposed in this research.

More significantly, the transconductance increased dramatically for the ET devices, strongly suggesting that the device mobility is much higher in the ET samples. The mobility can be extracted from the following relationship,

$${\mu }_{FE}=\frac{dI}{d{V}_{G}}\frac{{L}_{ch}}{{W}_{ch}}\frac{1}{{C}_{ox}{V}_{DS}},$$

where *μ*_*FE*_ is graphene field-effect mobility, ${L}_{ch}$ and *W*_*ch*_ are channel length and width, respectively, and ${C}_{ox}$ is oxide capacitance per unit area. In Fig. 3, the measured hole mobility reached *μ*_*h*_ = 1000 cm^2^/Vs for the AO device, and, in the case of the AA device, the hole and electron motilities reached as high as *μ*_*h*_ = 2580 cm^2^/Vs and *μ*_*e*_ = 1590 cm^2^/Vs, respectively. However, those in the ET sample in Fig. 3(c) marked as high as *μ*_*h*_ = 12440 cm^2^/Vs and *μ*_*e*_ = 12640 cm^2^/Vs, for the hole and electron carriers, respectively. The significant increase in the mobility upon the removal of the PMMA can be attributed to the decrease in carrier scattering^23^.

**Figure 4:**
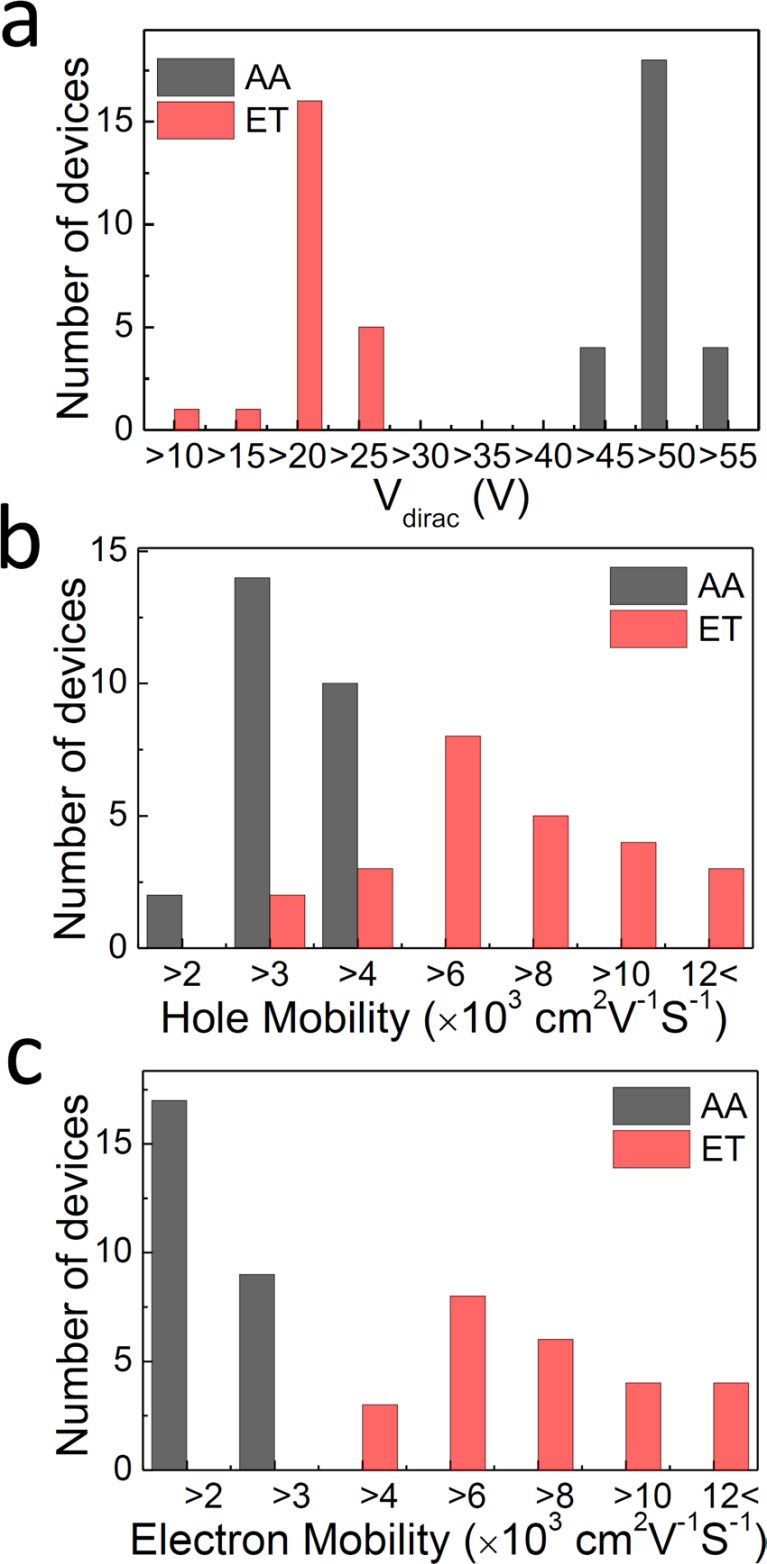
Histogram of (**a**) Dirac points, (**b**) hole mobility, (**c**) electron mobility of graphene FETs for AA (grey) and ET (red) samples.

The transport properties from more than 20 devices were each measured for the different groups of samples and the results for AA and ET samples are summarized in Fig. 4. Firstly, the Dirac points between the two device groups were compared, showing a decrease from 47.0 V (AA) to 17.6 V (ET) on average. Additionally, the Dirac point for AO sample was 74.1 V on average, however this is not shown. At this point, it was possible to estimate the effective surface charge density (*q*_eff_) responsible for the voltage shift (${\rm{\Delta }}{V}_{G}$) induced by PMMA in both the AO and AA cases, relative to the ET case. This was performed using the relationship ${q}_{eff}={C}_{ox}{\rm{\Delta }}{V}_{G}$, and oxide capacitance value ${C}_{ox}$ = 15.7 nF/cm^2^ (oxide capacitance was calculated using the relationship ${C}_{ox}={\varepsilon }_{ox}/{t}_{ox}$, where ${\varepsilon }_{ox}$ and ${t}_{ox}$ are the permittivity and the thickness of gate dielectric, respectively). As a result, it was estimated that there was excessive surface charge densities of 890 nC/cm^2^ and 460 nC/cm^2^ for the AO and AA devices respectively.

The increase in field-effect mobility is illustrated in Fig. 4(b) and (c) for the electron and hole cases, respectively. As can be seen in this figure, the hole mobility reaches 6770 cm^2^/Vs for the ET devices on average, double that of AA devices (2850 cm^2^/Vs) and seven times that of the AO devices (910 cm^2^/Vs, not shown). Similarly, electron mobility is shown to be as large as 7350 cm^2^/Vs for the ET devices, four times that of AA devices (1850 cm^2^/Vs). Surprisingly, the maximum values obtained in this research yields were as large as ~17000 cm^2^/Vs (similar for both electron and hole), which is unprecedentedly high for the CVD-grown graphene, among those measured in the ambient condition.

Finally, we summarize in Table 1 the mobility values obtained in our work, in comparison to those found in the literatures. Besides chemical treatment, there have been increasing efforts to obtain the large mobility seen by removing the PMMA residues effectively. Among the techniques developed to do this, UV treatment on the surface has proven to be very effective (*μ*_*h*_ ~ 4700 cm^2^/Vs), however, these techniques could either induce defects to the graphene or suffer from the lack of reproducibility^25,33^. Conversely, the techniques proposed in this research are based on the common knowledge that electron bombardment is best suited for removing the PMMA by its nature. It has been suggested that the entangled polymer chain on the graphene cannot be easily removed by the conventional solvents^26^; however, electron beam irradiation will induce the scission of the polymer chains, resulting in the effective removal of the PMMA residues^26^. In particular, our method has the advantage of removing the interaction between PMMA and the graphene surface, which is free from creating additional defects and contaminants. Thus, it was beneficial in helping to obtain a higher carrier mobility and move the Dirac point closer to a zero-gate bias even in the ambient condition. In addition, our technique requires relatively short periods of time and provides high mobility values.

**Table 1:** Comparison of the carrier mobility of graphene according to treatment methods of PMMA.

Treatment methods	Mobility (cm^2^V^−1^s^−1^)		Reference
	Hole	electron	
Automatic transfer	1500		^15^
AFM contact-mode	870	1200	^42^
Chloroform treatment	3100	2700	^25^
UV treatment	4700 (MAX)		^34^
Acetic acid treatment	2850	1850	This work
E-beam treatment	6770	7350	This work
(our maximum result)	(16980)	(16590)	

## Conclusion

In conclusion, an efficient technique was developed for removing PMMA layers at the graphene surface using electron beam bombardment. In this research, the FETs were fabricated with improved electrical properties on the transferred graphene after e-beam treatment. For instance, the Dirac point of the devices fabricated from this method was located very close to the zero-gate bias, in comparison to those from the acetone and the acetic acid treatments. Increased 2D peak in Raman spectra for the e-beam processes confirms the effective removal of the PMMA residues. More significantly, the field-effect mobility reached as high as 6770 cm^2^/Vs and 7350 cm^2^/Vs on average for holes and electrons, respectively, with the maximum values of up to 17000 cm^2^/Vs for hole mobility. This is greater than a seven time improvement in comparison to conventional acetone treatment, in terms of the average value, which is superior to other approaches reported for CVD grown graphene devices. Our work will open the door to the development of optimal procedures for fabricating future functional devices based on various two-dimensional materials.

## Methods

### Graphene synthesis and transfer

Graphene was prepared utilizing the CVD method on a 25 µm Cu foil (Alfa Aesar, No.13382) using methane and hydrogen gases. PMMA was employed as a graphene carrier from the Cu foil onto a Si substrate with an oxide layer of thickness 220 nm and heavily doped p-type Si layers of thicknesses 550 µm (the resistivity was 0.001–0.003 Ω/cm). The Si substrate contained the drain and source electrodes (Cr/Au) fabricated previously with conventional lithography techniques. After the Cu foil was fully dissolved in an ammonium persulfate solution, the PMMA/graphene film was transferred onto the silicon substrate.

### PMMA removal processes

After the transfer process, the PMMA layer was removed using electron-beam bombardment (ET process), followed by the developing processes. An electron-beam, with the areal dose of 100 μC/cm^2^ and current of 500 pA at 25 kV, was irradiated for 20 s through the region (50 × 200 μm^2^) that encompasses more than 6 devices. The exposed region of the PMMA was then removed by the developing process, which featured methyl isobutyl ketone (MIBK) 3:1 solution and lasted for 3 minutes. After the developing, PMMA residues were removed thoroughly by dipping the sample in acetone solution for 2 hours. Besides from the ET process, two conventional processes (AO and AA processes) were added for comparison. For the AO process, the sample was submerged in the acetone solution for 24 hours at room temperature. Conversely, during the AA process, the sample was dipped in acetone solution for 1 hour and then immediately immersed in acetic acid for 24 hours at room temperature. After the PMMA removal processes (ET, AO, and AA processes), graphene patterns were generated (width ~1 µm and channel length of 7–10 µm) using the electron-beam lithography technique with negative e-beam resist (Ma-N 2401), followed by reactive ion etching.

### Raman measurements

Our homemade micro-Raman spectroscopy equipment consists of a 532 nm DPSS laser, spectrometer (Andor Shamrock 303i), and CCD (Andor iDus401A). An objective lens (20X, N.A = 0.75) was used to focus the laser with resolution of ~1 μm with the laser intensity at 10 mW.

## Electronic supplementary material

Supplementary Information
