# Supplementary material for: Electron beam induced removal of PMMA layer used for graphene transfer
Source: Sci Rep. 2017 Dec 22;7:18058. doi: 10.1038/s41598-017-18444-1 (PMC5741774; doi:10.1038/s41598-017-18444-1)
Supplement: Supplementary file 1 — Supplementary Information [file 41598_2017_18444_MOESM1_ESM.docx]

**Supplementary Information**

Electron beam induced removal of PMMA layer used for graphene transfer

*B. H. Son, H. S. Kim, H. Jeong, Ji-Yong Park, Soonil Lee, and Y. H. Ahn**

*Department of Physics and Department of Energy Systems Research, Ajou University, Suwon 16499, Korea*

*Corresponding author. Electronic mail: ahny@ajou.ac.kr

**S1. Effects of PMMA residues on Raman signals**


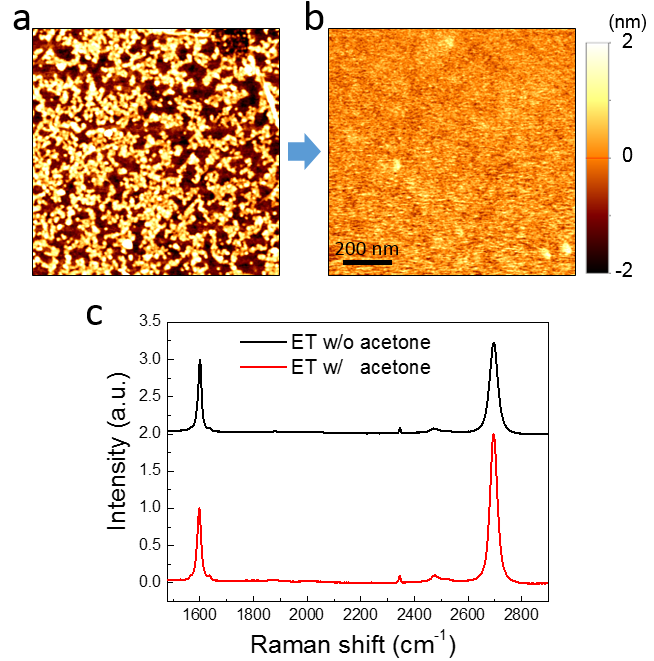


**Figure S1.** (a) An AFM image of the transferred graphene films after the PMMA has been removed by the e-beam irradiation and the following developing process with methyl isobutyl ketone (MIBK) 3:1 solution for 3 min. Without the additional acetone solvent treatment, the significant PMMA residues were left as clearly visible in the image. It is clearly demonstrated that the developing processes of 3 min is not enough to remove the PMMA residues thoroughly. (b) An AFM image of the film after dipping the graphene film of (a) in acetone solution for 2 hours. The PMMA residues have been largely removed in the image. (c) Raman spectra before (black line) and after the acetone treatment (red line), which correspond to the samples in (a) and (b), respectively. The intensity ratio of $I_{2D}/I_{G}$ increased from 1.22 to 2.00 after removing the PMMA residues by the addition acetone treatment. Importantly, this result confirms that the removal of the PMMA result in the increase in $I_{2D}/I_{G}$ due to the reduced doping effects.
